# Multipurpose Drugs Active Against Both *Plasmodium* spp. and Microorganisms: Potential Application for New Drug Development

**DOI:** 10.3389/fcimb.2021.797509

**Published:** 2021-12-24

**Authors:** Takuro Endo, Hitoshi Takemae, Indu Sharma, Tetsuya Furuya

**Affiliations:** ^1^ Laboratory of Veterinary Infectious Diseases, Cooperative Department of Veterinary Medicine, Faculty of Agriculture, Tokyo University of Agriculture and Technology, Tokyo, Japan; ^2^ Center for Infectious Disease Epidemiology and Prevention Research, Faculty of Agriculture, Tokyo University of Agriculture and Technology, Tokyo, Japan; ^3^ Department of Biological Sciences, Hampton University, Hampton, VA, United States

**Keywords:** drugs, malaria parasites, antimicrobial, repurposing, viruses

## Abstract

Malaria, a disease caused by the protozoan parasites *Plasmodium* spp., is still causing serious problems in endemic regions in the world. Although the WHO recommends artemisinin combination therapies for the treatment of malaria patients, the emergence of artemisinin-resistant parasites has become a serious issue and underscores the need for the development of new antimalarial drugs. On the other hand, new and re-emergences of infectious diseases, such as the influenza pandemic, Ebola virus disease, and COVID-19, are urging the world to develop effective chemotherapeutic agents against the causative viruses, which are not achieved to the desired level yet. In this review article, we describe existing drugs which are active against both *Plasmodium* spp. and microorganisms including viruses, bacteria, and fungi. We also focus on the current knowledge about the mechanism of actions of these drugs. Our major aims of this article are to describe examples of drugs that kill both *Plasmodium* parasites and other microbes and to provide valuable information to help find new ideas for developing novel drugs, rather than merely augmenting already existing drug repurposing efforts.

## Introduction

Based on the publication from the World Health Organization (WHO), there were 229 million sick people and 409,000 deaths including 67% of children aged under 5 years old, due to malaria in the world in 2019 (World Malaria Report, 2020). Despite the size of such severe damages, malaria distribution is very much skewed in a small number of regions; 94% of the cases are in the African region, followed by 2.5% and 2.2% in the Eastern Mediterranean and Southeast Asian regions, respectively, which results in disproportional burdens on the people in such areas regardless of the area’s net populations (World Malaria Report, 2020). Among the five *Plasmodium* spp. which infect humans, *Plasmodium falciparum* has the highest pathogenicity and accounts for most of the cases in the African region (World Malaria Report, 2020). Other than the regular malaria symptoms such as fever, aches, and weakness, *P. falciparum* can cause complicated malaria symptoms including respiratory distress caused by acidosis, pulmonary edema and pneumonia, neurological symptoms (cerebral malaria), enlarged spleen and liver, and hematuria, all of which can lead to unconsciousness followed by death (Muradi et al., 2015). Also, *P. falciparum* can cause serious problems in infected pregnant women (pregnant malaria). These serious symptoms are the results of sequestration of the parasite on the epithelial cells in the microvasculature, which makes *P. falciparum* the most virulent among the human malaria parasites (Muradi et al., 2015).

Regarding chemotherapeutic treatment of malaria, the drug resistance of the *Plasmodium* spp. has been one of the most serious problems necessary to be addressed. Drug-resistant *P. falciparum* have emerged for virtually all antimalarial drugs, including chloroquine, quinoline, sulfadoxine–pyrimethamine, mefloquine, or quinoline, and also artemisinin (ART), the essential drug in the current first-line treatment of ART combination therapies (ACTs) (White, 2004; Menard and Dondorp, 2017; World Malaria Report, 2020). To cope with the current situations of the *P. falciparum* drug resistance, the development of new antimalarial drugs has been one of the priorities in the malaria research communities, leading to the discovery of some new candidate drugs (Tilley et al., 2016).

Antiviral chemotherapeutics have been one of the subjects which have been focused in the field of public health. Transboundary infectious diseases are among the most serious issues not just in developing countries but also in economically developed countries and include diseases caused by human immunodeficiency virus (HIV), pandemic H1N1 influenza A virus, Zika virus, Ebola virus, dengue fever virus, West Nile virus, severe acute respiratory syndrome (SARS) virus, Middle East respiratory syndrome (MERS) virus, and currently, coronavirus disease 2019 (COVID-19) caused by severe acute respiratory syndrome coronavirus 2 (SARS-CoV-2) infection. Although there have been developments of some antiviral drugs in practice such as adamantane derivatives and neuraminidase inhibitors for influenza A virus (Paules and Subbarao, 2017) and a group of antiretroviral therapy drugs for HIV (Saag et al., 2018), practically, no other effective antiviral drugs have been developed yet.

Repurposing of already approved drugs for drug treatment different from the original purpose is an alternative approach compared with the discovery of entirely new drugs and often beneficial since the toxicity risk of the drug can be eliminated and the required time and costs for the required human trial processes can be substantially reduced; the average cost for a repurposed drug is US$300 million, compared with an estimated $2–3 billion for a new chemical entity with success rates of less than 4% in the top 3 areas of drug development between 1990 and 2004 (Pammolli et al., 2011; Pushpakom et al., 2019). However, while the large number of success cases exists, there are also some chance of failure in the trials and some other potential reasons for failure such as reasons related to patents, regulatory issues, or organizational problems (Pushpakom et al., 2019), and recent examples of failure of repurposed drug development were caused by failures in phase III trials or lack of efficacy in the cohort study (Pushpakom et al., 2019).

In the current situation of COVID-19, the antiviral effects of some antimalarial drugs have been drawing attention, leading to an increase of related studies (Rodrigo et al., 2020; Rakedzon et al., 2021). Also, considering the recent surge of antiviral drug development, increased reports of antimalarial activities of those drugs may be expected and vice versa. For this reason and to take advantage of such current trends, in this article, we focus on the drugs with multipurpose activities, aiming at not just contribution for finding effective drugs for repurposing and multipurpose use, but rather providing information to help find effective new ideas of novel drug development based on the known mechanisms of actions which make multipurpose activities of these drugs, including redesigning the existing drugs. Moreover, to aid this purpose, we chose the multipurpose drugs whose drug mechanisms have been relatively well studied. We also describe some antibacterial and antifungal drugs and some compounds found from marine sponge that possess multipurpose antimalarial activities.

## Antimalarial Drugs With Antiviral Activities

### Chloroquine

#### Antimalarial Action of Chloroquine

Chloroquine (CQ) is an FDA-approved drug used for the treatment of malaria for decades (Goel and Gerriets, 2021). Although it was once used as a first-line drug against *P. falciparum*, the spread of drug-resistant parasites in the world forced halting its use in practice. The antimalarial action of CQ is related to hemoglobin digestion of malaria parasites. CQ exerts its antimalarial activity by inhibiting hemoglobin digestion in the digestive vacuole (DV) in the parasite, a lysosomal acidic organelle, in which the parasite digests hemoglobin transferred from the host red blood cell to heme and converts into non-toxic hemozoin (Coronado et al., 2014). CQ is a weak base and mostly exists as a protonated form (CQ+ or CQ++), while only small portion of unprotonated CQ can penetrate the cytoplasm, diffuse into the DV, and then becomes protonated to be accumulated in the DV. In the DV, CQ is thought to bind to heme and prevent hemozoin formation (Chinappi et al., 2010). Due to high effectiveness and availability, the use of CQ gained tremendous success and was heavily applied in the malaria treatments in the world, until the drug-resistant *P. falciparum* emerged in the late 1950s and spread throughout the world in the 1980s when morbidity and mortality were resurged since alternative drugs with similar effectiveness and availability to CQ were not available (Wellems and Plowe, 2001).

The resistance mechanism of CQ-resistant *P. falciparum* has been studied (Ross and Fidock, 2019). Inside the *P. falciparum* cell, CQ accumulates in an organelle, the DV, where hemoglobin is digested and incorporated with a toxic by-product of heme into non-toxic hemozoin, forming hemozoin crystals (Ross and Fidock, 2019). CQ is supposed to kill *Plasmodium* parasites by inhibiting the crystallization of heme into hemozoin inside the DV. The major mechanism of CQ resistance of *P. falciparum* is the introduction of mutations inside or near the membrane-spanning regions of the *P. falciparum* chloroquine-resistant transporter (PfCRT) on the DV, which interferes CQ concentration inside the DV (Ross and Fidock, 2019). Though it affects CQ resistance to a less extent, the sequence variations in PfMDR, an ABC transporter on the DV membrane, can change the extent of CQ resistance of *P. falciparum* (Ross and Fidock, 2019).

#### Antiviral Action of Chloroquine

The antiviral activities of CQ have been also reported (Savarino et al., 2003a; Naghipour et al., 2020), and recently, CQ re-attracted attention for its antiviral activities, as COVID-19 caused problems across the globe. COVID-19 is caused by SARS-CoV-2 infection, a virus with a positive single-stranded RNA genome, and its major symptoms are similar to those of the common cold such as fever, body aches, cough, breathing problems, and shortness of breath, although people aged more than 60 years old and/or those with chronic health issues including diabetes, asthma, heart diseases, and obesity have higher chances of severe illness and resulting deaths (Pahan and Pahan, 2020). Initially, CQ was approved for the treatment of COVID-19 due to its *in vitro* antiviral activity (Wang et al., 2020). However, the low clinical efficacy rates of CQ became evident and the recommendation of CQ for COVID-19 treatment was withdrawn in the middle of 2020 (Mahase, 2020). Despite the outcome about its clinical use for COVID-19 problems, the antiviral activity of CQ has been reported and mounting evidence showed that it was associated with the accumulation of drugs in acidic organelles like the endosomes, Golgi vesicles, and lysosomes; the weakly basic accumulation of CQ in these acidic organelles led to the disruption of internal enzyme activities and to changes in the glycosylation of both the viral proteins and the counterpart receptors (Savarino et al., 2003b).

The inhibition of CQ in the glycosylation of the viral receptor was reported. Anti-SARS-CoV-2 activity was attributed to the inhibition of CQ of glycosylation of the viral receptor, angiotensin-converting enzyme 2 (ACE2), leading to interference in the binding of ACE2 and the spike (S)-protein of SARS-CoV-2 upon the virus entry in the host cells (Vincent et al., 2005; Chen et al., 2020). Such inhibition of glycosylation on ACE2 with CQ prevented SARS-CoV-2 from host cell infection, even in the cells treated with CQ prior to the virus inoculation (Chen et al., 2020). This study also demonstrated that SARS-CoV-2 infection was blocked by CQ when the cells were inoculated first and treated with the drugs 3–5 h later, which suggests that pH increase by CQ in the endosome could disrupt the fusion between viral and endosome membranes (Chen et al., 2020). Another study demonstrated the specific inhibition of CQ of quinone reductase 2 which is involved in the biosynthesis of sialic acid, a known binding molecule on the receptors of *P. falciparum* and other viral pathogens including ACE2, suggesting the association of this sialic acid synthesis by CQ with its broad range of antiviral activities (Chen et al., 2020).

Another potential mechanism of the antiviral activity of CQ is through changing the structures of viral envelope proteins. As a causative agent of AIDS, HIV, a member of the genus *Lentivirus* in the family *Retroviridae*, binds to its target CD4^+^ T cells through binding of the gp120 protein of the virus to the CD4 molecule, and loss of infectivity with the virus collected from HIV-infected cells with CQ treatment was reported in comparison to the virus from the untreated cells, suggesting that CQ changed the structure of HIV gp120 which bound to the host cell receptor (Savarino et al., 2001). Based on the result, a mechanism of action was proposed in which CQ changed the glycosylation of gp120 by inactivating glycosyl-transferases located in the Golgi complex. There may be other viruses in which CQ changes the structure of the virus proteins so that the virus loses its infectivity like HIV, since many viruses use glycoproteins for their attachment to their host cell receptors.

It is worth mentioning that CQ has anti-inflammatory and immunomodulatory effects to treat some autoimmune diseases, such as rheumatoid arthritis and systematic lupus erythematosus (Grygiel-Górniak, 2021), and some antiviral activities of CQ in *in vivo* assays and clinical trials might be affected by these activities on the immune system. Since CQ affects the glycosylation of proteins of both the host receptors and binding proteins of the viruses, it can exhibit its antiviral effects through both sides. On the other hand, such ability of CQ can also affect the immune functions of the infected humans, which should be considered when CQ is used for its antiviral effects.

### Artemisinin and Ivermectin

#### Antimalarial Action of Artemisinin

The selective toxicity of artemisinin (ART) and its fast-acting killing of the intraerythrocytic stages of *Plasmodium* spp. are derived from activation of the drug inside the parasites (Tilley et al., 2016). The endoperoxide bridge of ART reacts with reduced iron to be activated to generate radical species (Xie et al., 2020). The erythrocytic stage of malaria parasites uptakes the host hemoglobin through ingestion at the parasite surface structures known as cytostomes and hemoglobin-containing cytostomal invaginations fuse with an acidic DV. Inside the DV, hemoglobin is digested, and free hemes and free ferrous iron (Fe^2+^) released through the digestion reactions cleave the endoperoxide bridge of ART. Only after this cleavage of the endoperoxide bridge, ART exhibits its antimalarial activity with the carbon-centered free radicals produced by the endoperoxide bridge cleavage (Tilley et al., 2016). Activated ART reacts with susceptible groups (nucleophiles) in proteins, lipids, and heme, leading to widespread cellular damage (Xie et al., 2020). Recent studies suggest that ART also causes generalized unfolding and damage to proteins and blocks the proteasome protein degradation, which leads to prolonged and unresolvable stress, killing the parasite (Bridgford et al., 2018). Also, some studies suggested that ART-induced reactive oxygen species (ROS) was involved in the rapid killing of *P. falciparum* through depolarization of the parasite mitochondrial and plasma membranes upon exposure to ROS (Antoine et al., 2014; Bridgford et al., 2018; Connelly et al., 2021). The quick action of ART due to its drug action mechanism is a strong advantage of the drug. However, this advantage also makes the half-life of ART especially short (~1 h in humans), which makes ART monotherapy impossible and the need of other drugs with long half-lives for the ACT (Tilley et al., 2016).

The emergence of ART-resistant *P. falciparum*, manifested by slower rates of parasite clearance, was reported in 2014 in South Asian countries (Ashley et al., 2014), and mutations in the Kelch13 (K13)-propeller protein was reported as the major driver for the ART-resistant phenotype (Ariey et al., 2014). One member of the superfamily of Kelch-repeat protein, which the K13-propeller protein belongs to, human KEAP1, binds to ubiquitin ligase E3 and a ubiquitination substrate transcription factor at each binding site (Tilley et al., 2016), suggesting that the K13-propeller protein of *P. falciparum* may also function as an adapter protein that controls the nuclear binding of a stress response transcription factor through ubiquitination and proteosome degradation (Tilley et al., 2016). Sequencing of parasite strains revealed that mutations in other proteins, such as apicoplast ribosomal protein S10, multidrug-resistance protein 2, ferredoxin, and PfCRT, provided conditions that led to K13 mutations (Ariey et al., 2014). In addition, a notable copy number change of *pfmdr1* was reported to be involved in ART resistance (Veiga et al., 2016).

#### Antiviral Action of Artemisinin

ART and its derivatives were reported to have *in vitro* antiviral activity at least against the human cytomegalovirus (HCMV), hepatitis B virus (HBV), hepatitis C virus (HCV), HIV, and SARS-CoV-2 (Romero et al., 2005; Oguariri et al., 2010; Obeid et al., 2013a; Oiknine-Djian et al., 2018; Cao et al., 2020). HCMV is a ubiquitous species of betaherpesvirus with a double-stranded DNA genome and can cause diseases in infected patients whose immune systems are compromised such as AIDS patients or recipients of organ transplants or hematopoietic stem cells, while HBV, a member of the family *Hepadnaviridae* with a circular partially double-stranded DNA genome, can cause acute and chronic liver diseases which may develop into liver cancers, and HCV, a member of the family *Flaviviridae* with a single-stranded RNA, causes chronic infections that can develop into cirrhosis or liver cancers (Romero et al., 2005; Oguariri et al., 2010; Obeid et al., 2013a).

The antiviral mechanism of ART is yet to be fully understood and possibly different among virus species. One of such potential mechanisms is the one through induction of ROS by ART, but not through carbon-centered free radicals like the one for its antimalarial activities. One study showed that 50% maximal effective concentration (EC_50_) of ART against HCV-infected cells was 2- to 5-fold reduced by the treatment with L-N-acetylcysteine (L-NAC), which inhibits free radical generation (Obeid et al., 2013b).

Another mechanism of antiviral effect of ART was proposed with HCMV infection model, in which ART inhibited the virus replication by interfering the host cell signaling necessary for the virus replication. Interference of the nuclear factor kappa B (NF-kB) pathway by targeting RelA/p65 was reported as a mechanism of artesunate action on HCMV-infected cells (Hutterer et al., 2015). RelA/p65 is a member of the NF-kB family, which normally induces antiviral interferon responses upon virus infections but also in the early stage of the infection, and stimulates HCMV major immediate early promoter (MIEP) to enhance HCMV replication. Therefore, ART exhibits anti-HCMV activity by inhibiting the NF-kB pathway (Hancock and Nelson, 2017). In addition, other studies suggested that ART inhibition in the PI3K/Akt signaling pathway could be a Target of antiviral action of the drug. ART inhibition of the PI3K/Akt pathway was reported in the study of HCMV-infected cells, in which activations of Akt and p70S6K, signals in downstream of the PI3K/Akt pathway and essential for HCMV replication, were inhibited with ART (Efferth et al., 2002). PI3K/Akt is one of the master genes that regulate many other genes, including the essential genes in the processes necessary for cell maintenance, glucose metabolism, protein synthesis, and cell cycle and growth (Hemmings and Restuccia, 2012). Activation of Akt is also essential for HCMV DNA replication, which was blocked when this pathway was inhibited with ART treatment (Johnson et al., 2001).

The antiviral effect of ART was also reported against SARS-CoV-2 in an *in vitro* study (Zhou et al., 2021a). This action can be through inhibition of the PI3K/Akt pathway as the case with HCMV since SARS-CoV-2 utilizes host clathrin-mediated endocytosis upon the host cell invasion and this process is regulated by the PI3K/Akt pathway (Khezri, 2021). On the other hand, a molecular docking study reported that ART could physically bind to the receptor binding domain (RBD) of the SARS-CoV-2 spike protein and interfere RBD binding to hACE2 (Sehailia and Chemat, 2021). This report suggests that ART may exhibit anti-SARS-CoV-2 activity by interfering the entry of the virus with its competitive interaction with the viral receptor (Sehailia and Chemat, 2021). Despite this report, SARS-CoV-2 infection to the culture cells was not inhibited by the addition of artesunate at the time of inoculation, suggesting that ART action against SARS-CoV-2 was active only after the virus entry into the host cells (Zhou et al., 2021b).

Since the proposed mechanisms of ART against viral pathogens such as the production of ROS or inhibition of the signaling pathways are different from the ones against the malaria parasites, it may not have the same disadvantage of extremely short half-life in the host when used for antiviral purpose. At the same time, ART may disturb the host immune functions since some of the inhibited signal pathways are shared with those in immune cells (Khezri, 2021).

#### Antiviral Activity of Ivermectin

Another interesting drug mainly used for the treatment of helminth infections but also active on *Plasmodium* spp. is ivermectin. Ivermectin is used for the treatment of nematode parasitic diseases, such as onchocerciasis and lymphatic filariasis (Crump and Ōmura, 2011). The antiparasitic mechanism of ivermectin on helminths including nematodes and insects is binding to glutamate-gated chloride channels in invertebrate muscles and nerves, which leaves the channels open leading to a flow increase of the chloride ions and subsequent hyperpolarization of the cell membranes and paralyzes and kills the parasites (Shiomi, 2021). Studies demonstrated the activity of ivermectin against hepatic stages, erythrocytic stages, sexual stages, and oocyst and sporozoite stages of *Plasmodium* spp (Kobylinski et al., 2012; Mendes et al., 2017; Azevedo et al., 2019; de Carvalho et al., 2019). The mechanism of antimalarial action of ivermectin was reported to be the specific inhibition of the importin α/β1-mediated nuclear import (Panchal et al., 2014; Oany et al., 2021).

Studies reported the antiviral activity of ivermectin against HIV (Wagstaff et al., 2012a), flaviviruses (such as dengue, Zika, yellow fever, and West Nile viruses) (Mastrangelo et al., 2012), influenza A virus (Götz et al., 2016), and SARS-CoV-2 (Caly et al., 2020). The proposed mechanism of action against these viruses is through inhibition of nuclear transport mediated by the importin α/β1 heterodimer (Wagstaff et al., 2011; Wagstaff et al., 2012b). Though most of the studies of antiviral activities of ivermectin were based on assessments with *in vitro* assays, some studies on its effect on SARS-CoV-2 patient treatment were performed based on a small-scale clinical trial (Pott-Junior et al., 2021; Zhang et al., 2021) or meta-analyses of the publications and databases (Deng et al.,; Cruciani et al., 2021) about its effects on prophylaxis or prognosis of the treatments, which were mixed in terms of the efficacy of the drug on its clinical use and none was conclusive, along with negative results with *in vitro* study (Dinesh Kumar et al., 2021), stressing the need for more studies about its clinical application.

The antiparasitic mechanism of artemisinin is not well understood despite some are proposed as above, in contrast to its well-understood mechanism for the parasites, which is one of the hurdles of the drug for its practical use for patients with viral pathogens (Dinesh Kumar et al., 2021).

## Antiviral Drugs Active Against *Plasmodium* spp.

### Antiretroviral Protease Inhibitors

Since coinfection with HIV is often common in malaria endemic areas, drugs active against both HIV and *Plasmodium* spp. are demanded for the people in such areas. One such drug is antiretroviral protease inhibitors (ARPIs), which are known to inhibit the growth of malaria parasites (Parikh et al., 2005). ARPIs are transition state analogs that inhibit viral aspartic protease essential for viral maturation. HIV expresses Gag-Pol polyprotein whose cleavage is essential for the virus replication (Konvalinka et al., 2015). Studies reported some ARPIs with the ability to enhance the antimalarial activity of artemisinin and chloroquine (Mishra et al., 2010; Li et al., 2011). One of the ARPIs, indinavir, appeared to have synergic activity with CQ in *in vitro* and *in vivo* assays (Li et al., 2011). In *in vivo* assay with *P. chaboudii*, co-treatment of the infected mice with indinavir and CQ cleared substantially higher percentages of the parasite than the ones treated with either drug alone from both the CQ-sensitive and CQ-resistant parasites, ASS and ACQ strains, respectively (Li et al., 2011).

While the antimalarial mechanism of ARPIs has not been understood yet, some studies suggest plasmepsins and its homologous proteases as important targets of ARPIs for *P. falciparum* and other *Plasmodium* spp., respectively. The results of *in silico* docking analysis supported the bindings of ARPIs (saquinavir, ritonavir, and lopinavir) with the active sites of *P. falciparum* plasmepsins, multiple-copy aspartic proteases involved in hemoglobin digestion of the parasite, suggesting potential targets of ARPIs against *Plasmodium* spp. in their hemoglobin digestion pathways which are essential for the antimalarial activities of both ART and CQ (Andrews et al., 2006). In support of this finding, gene-disrupted mutants for each of the four food-vacuole-localizing plasmepsins were produced, and two of them, gene-knockout mutants for PfPM1 or PfPM4, displayed substantially slower growth, indicating the important roles of plasmepsins for the growth of intraerythrocytic stages of *P. falciparum*, which was in agreement with the model in which plasmepsins are the target molecules of ARPIs (Omara-Opyene et al., 2004).

The other potential mechanism of the antimalarial activity of ARPIs is the reduction of the amount and activity of the antioxidants glutathione (GSH) and glutathione S-transferase (GST), respectively, in *Plasmodium* parasites (He et al., 2009). In this study, higher levels of GSH and GST were observed in the CQ-resistant strains of *P. falciparum* and *P. chabaudi* than the sensitive ones, and the induction of those chemicals in the CQ-resistant parasites was lower when treated with ARPIs (saquinavir, ritonavir, and nelfinavir) than the untreated ones (He et al., 2009). Since detoxication of heme in the parasites is essential for the survival of *Plasmodium* spp., the reduction by ARPIs of GSH and GST, which detoxify heme made from hemoglobin digestion, may explain the mechanism of the synergizing effects of ARPIs on CQ or ART antimalarial activities.

Since ARPI specifically inhibits the protease of HIV, its simultaneous inhibition of plasmepsins of the malaria parasites is beneficial since co-infections of HIV and *Plasmodium* spp. are quite common in endemic areas (Andrews et al., 2006). On the other hand, the effect of ARPI against malaria parasites may be limited since *Plasmodium* spp. express multiple copies of plasmepsin genes and homologs, suggesting that the simultaneous use of another antimalarial drug may be essential in the practice of malaria treatment (Omara-Opyene et al., 2004).

### Amantadine (Anti-Influenza A Virus)

Amantadine is a drug used for the treatment of influenza A virus infection. With a large number of deaths with infected patients every year, the influenza A virus is an enveloped virus with the segments of single-stranded, negative-sense RNA genome, which causes the most severe diseases among the four influenza virus types and infects a variety of animals such as humans, pigs, horses, and bird species, and therefore, becomes sources of serious zoonotic infections (Kumar et al., 2018). Amantadine blocks the M2 protein, a transmembrane proton channel on the virus particle, to inhibit proton flow into the virus which is required for the uncoating of the virus (Kausar et al., 2021). Amantadine exhibited inhibition of *P. falciparum* growth, which was strain-specific and more prominent with CQ-resistant strains in general; the *in vitro* IC_50_ values were 5–50, 250, and 350–450 µM for the CQ-resistant Southeast Asian strain, CQ-resistant South American strain, and CQ-sensitive line, respectively (Evans and Havlik, 1996; Wellems, 2004). These differences may be explained with the characteristics of amantadine as a channel blocker (Johnson et al., 2004). CQ-resistant *P. falciparum* can efflux CQ from the food vacuole through the mutated PfCRT, while wild-type PfCRT is positively charged, and CQ, protonated and positively charged inside the food vacuole, cannot exit due to repulsion from the positively charged PfCRT, because the mutated amino acid residue, such as lysin at position 76, of PfCRT was replaced with allosteric threonine (Fidock et al., 2000). This mechanism was also supported by the effect that verapamil, a positively-charged calcium channel blocker that also blocks CQ exhaustion through PfCRT, bound to PfCRT, blocked CQ efflux, and restored CQ sensitivity only with mutated PfCRT but not with the wild-type PfCRT (Wellems, 2004). In addition, single amino acid mutation of serine to arginine at position 163 on PfCRT was found in amantadine-resistant *P. falciparum*, generated from a CQ-resistant Southeast Asian strain by growing under amantadine pressure, which restored the positive charge of the protein and inhibited amantadine binding (Johnson et al., 2004). The mechanism of amantadine, the inhibition of the proton channels of influenza A virus and *P. falciparum*, is different from the other drugs for these pathogens, which makes this drug useful for the treatment of the drug-resistant virus or parasites. On the other hand, its effect on *P. falciparum* is strain-dependent, and the antimalarial activity of amantadine can be low even for CQ-sensitive *P. falciparum* depending on the strain, and its use for malaria treatment may not be appropriate though it can be useful for some CQ-resistant *P. falciparum* strains (Evans and Havlik, 1996; Wellems, 2004).

## Antibacterial Drugs Active Against *Plasmodium* spp.

Antibacterial drugs have been widely used for the treatment of infectious diseases caused by bacterial infections. Among the antibacterial drugs, a number of antibacterial drugs target the bacterial cell wall (e.g., beta lactams, vancomycin, and bacitracin), while others target bacterial metabolic pathways (e.g., sulfonamides, trimethoprim), protein synthesis (e.g., macrolides, tetracyclines, aminoglycosides), and nucleic acid synthesis (e.g., quinolones, rifampin) (Kapoor et al., 2017). Due to the similarities in the protein structures and requirements of some metabolic pathways between bacteria and *Plasmodium* spp., some antibacterial drugs also have antimalarial activities (Kapoor et al., 2017). Here, we describe some of such examples and the potential antimalarial mechanisms of the drugs.

### Folate Synthesis Pathway Inhibitors

Inhibitors of folate synthesis pathway (FSP), such as sulfonamides, are widely used antibacterial agents and inhibit the synthesis of folate, a major precursor of nucleic acids of bacteria. Sulfadoxine–pyrimethamine (SP) targets dihydropteroate synthase (DHPS) and dihydrofolate reductase (DHFR) of *Plasmodium* spp., respectively, which are the enzymes catalyzing two reactions in the series in FSP (Triglia and Cowman, 1999; Hyde, 2005). Though it was once recommended by the WHO for malaria treatment, it is currently not used regularly due to the emergence of drug resistance but still used for pregnant women and infants [intermittent preventive treatment for malaria in pregnancy (IPTp)] (World Malaria Report, 2020).

Antimalarial activity was reported for another combination of FPS inhibitors, co-trimoxazole, a mixture of trimethoprim and sulfamethoxazole, through clinical studies of HIV patients (Mermin et al., 2006; Kamya et al., 2007; Sandison et al., 2011; Suthar et al., 2015). Daily administrations of co-trimoxazole to HIV-infected patients for prophylaxis against opportunistic infections resulted in lower rates of *Plasmodium* spp. infections compared with the untreated ones. Similar to SP, the targets for sulfamethoxazole and trimethoprim are the enzymes in the series of FSP, DHPS, and DHFR, respectively (Cockerill and Edson, 1991). Lower infection rates of bacterial infections can be expected when FSP is used for malaria treatments in areas where both infections are common, although the emergence of drug-resistant bacteria and *Plasmodium* parasites can occur with high frequencies, and the simultaneous use of other antibacterial and/or antimalarial drugs is necessary in case FSP is used for both of these pathogens.

### Tetracyclines

Tetracycline antibiotics (tetracyclines) are a group of antibiotics effective against a wide range of bacteria, including Gram-positive and Gram-negative bacteria, which have been used for the treatment and prophylaxis of *P. falciparum* and *Plasmodium vivax* (Pessanha de Carvalho et al., 2021).

Tigecycline is a member of the third-generation TC derivatives and in a class of tetracyclines, glycylcycline, which was developed to overcome multidrug-resistant bacteria (Yaghoubi et al., 2021). Based on *in vitro* assay with a clinical isolate of *P. falciparum*, the IC_50_ values for tigecycline and doxycycline were 699 and 4,276 nM, respectively, suggesting faster action of tigecycline than tetracyclines of the older generations (Starzengruber et al., 2009).

The target of tetracyclines in *Plasmodium* spp. could be unique compared with the ones in the sensitive bacterial species. The antibacterial action of tetracyclines is translation inhibition of the chromosomal genes by the drug binding to several proteins in the 30S ribosomal small subunit and to some different ribonucleic acids in the 16S rRNA (Gaillard et al., 2015). Multiple studies reported that the component of expression machinery for the genes contained in the genome of apicoplast, a unique plastid organelle of apicomplexan parasites, was the target of tetracyclines, and doxycycline directly inhibited the expression of genes in the apicoplast genome of *P. falciparum* (Dahl et al., 2006; Dahl and Rosenthal, 2007a; Chakraborty, 2016; Okada et al., 2020). Loss of apicoplast functions in the doxycycline-treated parasite led to delayed death in the second cycle (Dahl et al., 2006; Dahl and Rosenthal, 2007a). Although slow actions of many tetracyclines can be problematic for malaria treatments, the potentiation of tigecycline of CQ for CQ-resistant *P. falciparum* W2 strain was reported, while the mechanism of tigecycline for its faster action was not clear yet, suggesting the value of potential treatments using combinations of tigecycline and other antimalarial drugs to overcome the emergence of drug resistance (Sahu et al., 2014).

### Fosmidomycin

Fosmidomycin is an antibiotic derived from *Streptomyces lavendulae* (Iguchi et al., 1980; Parkinson et al., 2019). Fosmidomycin inhibits 1-deoxy-D-xylulose 5-phosphate (DOXP) reductoisomerase, which is associated with non-mevalonate pathway (MEP) in isoprenoid biosynthesis (Kuzuyama et al., 1998). MEP is utilized by Gram-negative bacteria, some Gram-positive bacteria, plastid-containing eukaryotes, and plants, while mammals use the mevalonate pathway (Rohmer et al., 1993; Lange et al., 2000; Armstrong et al., 2015). Apicomplexa parasites including *Plasmodium* spp. synthesize isoprenoides with MEP and harbor the DOXP pathway in apicoplast, a unique organelle that originated from algae through secondary endosymbiosis (Nair et al., 2011). The discovery of MEP in malaria parasites provided the potential target of new antimalarial compounds (Wiesner et al., 2003).

The effectiveness of fosmidomycin against malaria was examined with *in vitro* and *in vivo* assays as well as clinical trial. *In vitro* IC_50_ values for fosmidomycin were 819 and 926 nM with 3D7 and Dd2 strains of *P. falciparum*, respectively (Wiesner et al., 2002). In addition, fosmidomycin–piperaquine combination therapy for uncomplicated malaria was on phase 2 clinical trial performed in Gabon between 2014 and 2016 and demonstrated high efficacy (Mombo-Ngoma et al., 2018). Using fosmidomycin for malaria treatments can be an attractive drug choice since it targets an enzymatic pathway in a unique organelle of the apicoplast, though similar to other apicoplast-target antimalarial drugs such as tetracycline, a slower action compared with other antimalarials can be a consideration for its practical use.

### Macrolides

Macrolides are a class of natural products used to treat bacterial, viral, and parasitic infections (Lee et al., 2011; Dinos, 2017; Poddighe and Aljofan, 2020). In bacteria, macrolides bind to the neighbor of the entrance of polypeptide tunnel in 50S ribosomal subunit. This binding prevents transpeptidation of polypeptides, leading to immature peptide chain production and cell death (Retsema and Fu, 2001; Hansen et al., 2002; Schlünzen et al., 2003; Sidhu et al., 2007a). The efficacy of azithromycin against malaria parasites has been confirmed in *in vitro* and *in vivo* experiments and clinical trials (Andersen et al., 1994; Ohrt et al., 2002; Sagara et al., 2014). The target of azithromycin in *P. falciparum* was identified as 50S ribosomal subunit in apicoplast based on a study with azithromycin-resistant *P. falciparum*, the same target as the one for its antibacterial activity but in a unique organelle of apicomplexa parasites (Sidhu et al., 2007b). The delayed effect of azithromycin was documented in this study with IC_50_ values of 3,500 nm for 48 h of incubation and 103 nm for 96 h of incubation, more than 30 times difference at the delayed time point (Sidhu et al., 2007b). The delayed effect of azithromycin was explained by the effect of specific toxicity of the drug on apicoplast and death of the parasite in the second cycle due to lack of the apicoplast genome replication in the first cycle (Dahl and Rosenthal, 2007b). In addition to this delayed action, azithromycin demonstrated fast-killing activity which was independent of apicoplast-mediating delayed killing and augmented in azithromycin analogs with side-chain modifications, although the mechanism of this fast-killing mode of the drug was not understood (Burns et al., 2020). Similar to other drugs like tetracycline and fosmidomycin, azithromycin targets an enzymatic pathway in apicoplast, which makes the drug selective to *Plasmodium* parasites (Sidhu et al., 2007b) but makes the action of the drug slow for its disadvantage also (Dahl and Rosenthal, 2007b).

## Sponge-Derived Product

### Manzamines

Manzamines are β-carboline alkaloids isolated from marine sponge species. Manzamine A has been isolated from several sponge species such as *Haliclona*, *Pellina*, *Pachypellina*, *Xestospongia*, *Ircinia*, and *Amphimedon* (Ang et al., 2000). Manzamine A and 8-hydroxymanzamine A were reported to have *in vivo* antimalarial effect on *Plasmodium berghei*-infected mice (Ang et al., 2000).

Manzamines are inhibitors of glycogen synthase kinase-3 (GSK-3), which is also thought to be the target for their antimalarial activities (Skropeta et al., 2011); therefore, *P. falciparum* GSK-3 (PfGSK-3) was evaluated as a potential target of new antimalarial compounds. However, some studies reported high sequence identities between PfGSK-3 and its human homologs GSK-3α or GSK-3β (Droucheau et al., 2004) and the narrow therapeutic index of manzamine A with *P. berghei*-infected hosts (Ang et al., 2000); 50 to 100 μmol/kg of manzamine A prolonged the survival of infected mice, while the minimum toxicity concentration was 500 μmol/kg (Ang et al., 2000). On the other hand, paullons, another inhibitor of mammalian GSK-3β, exhibited the same percentage of PfGSK-3 inhibition with 100- to 300-fold fewer molar concentrations (Leost et al., 2000), suggesting that the structural characteristics of paullons may help find manzamines and their analogs or other chemicals with higher specific activities against PfGSK-3. The target of manzamines for its antimalarial activity is unique compared with other major antimalarials, while toxicity due to the similarity between enzymes of the malaria parasites and human can be a major disadvantage of the drug.

### Marine Isonitriles

The first marine isonitrile was found from the sponge *Axinella cannabina* (Emsermann et al., 2016). The antimalarial activity of several isonitriles was reported (Emsermann et al., 2016; Álvarez-Bardón et al., 2020). A docking study of some isonitriles with human hemoglobin suggested their bindings with heme, formations of a complex with heme iron, and subsequent inhibition of heme detoxication process as their antimalarial mechanism (Wright et al., 2001).

## Antifungal (Antimycotic) Drugs That Show Potential Against Malaria Parasites

Fungal pathogens are involved in various types of diseases, such as skin diseases and diseases on mucus membranes like the throat and female genitalia, and can be fatal when the infections spread to the whole body through the blood system (Crowley and Gallagher, 2014). The treatment can be problematic since fungal pathogens are eukaryotic organisms like human hosts, and many drugs that kill fungus are also toxic to humans, and antifungal drugs have to have drug mechanisms selective to the fungal pathogens despite the similarities as eukaryotes between fungi and humans. On the other hand, some antifungal drugs also kill malaria parasites due to their similarities to the fungi as they are both eukaryotic organisms (Crowley and Gallagher, 2014).

### Clotrimazole

Clotrimazole is an antimycotic drug used for the treatment of skin infections, such as *Candida albicans* and other fungal infections, by inhibition of the biosynthesis of ergosterol, a sterol found in fungi and protozoa (Crowley and Gallagher, 2014). Clotrimazole also inhibits *P. falciparum* growth in both chloroquine-resistant and chloroquine-sensitive strains *in vitro* (Tiffert et al., 2000). Inhibition of hemoperoxidase by clotrimazole leading to the subsequent induction of oxidative stress in *P. falciparum* was reported as the antimalarial mechanism of clotrimazole (Trivedi et al., 2005). Clotrimazole scaffold and its analogs were designed and synthesized as potent antimalarial agents (Gemma et al., 2007; Gemma et al., 2008). Clotrimazole nanoemulsion was formulated to improve its solubility (Borhade et al., 2012a; Borhade et al., 2012b), evaluated for its antimalarial activity using the *in vivo* assay with *P. berghei*-infected mice and confirmed to be significantly more effective in parasitemia inhibition and longevity of the infected host than clotrimazole suspension (Leost et al., 2000). Selectivity is one of the advantages of clotrimazole for its antimalarial activity due to its effect on the synthesis of ergosterols, which can be found mainly in fungi and protozoa (Crowley and Gallagher, 2014). Drug delivery to the place of pathogen multiplication can be an issue for its practical use.

### Griseofulvin

Griseofulvin is used to treat dermatophyte fungal infections, which binds to intracellular microtubules inhibiting mitosis of fungi (Lambert et al., 1989). Griseofulvin inhibited intraerythrocytic growth of *P. falciparum* in red blood cells pretreated with the drug *in vitro*, although no effect was observed in a human clinical trial for either therapeutic or prophylactic treatments (Smith et al., 2017). The antimalarial mechanism of action of griseofulvin was reported to be its cytochrome P450-mediated production of *N*-methyl protoporphyrin IX (*N*-MPP) and its inhibition of the heme-synthesizing ability of ferrochelatase, and despite its failure in clinical trial, this unique mode of action of griseofulvin demonstrated a new potential lead chemical to develop new antimalarial drugs (Smith et al., 2017).

### Ketoconazole

Ketoconazole interferes with ergosterol synthesis by inhibition of cytochrome P_450_ (CYP) 3A4 and is used to treat a number of fungal infections (Deutsch and Quintiliani, 1984). The combination treatment of ketoconazole and the artemisinin derivative α/β arteether demonstrated augmentation of antimalarial effect of α/β arteether with ketoconazole both with CQ-sensitive and CQ-resistant *P. falciparum* strains *in vitro* and with the multidrug-resistant *Plasmodium yoelii nigeriensis* in mice *in vivo* assay (Tripathi et al., 2013). Artemisinin and α/β arteether are known to be rapidly metabolized by the liver CYP, and ketoconazole inhibition of CYP 3A4 may slow down the α/β arteether metabolism and contribute to enhance the drug action by prolonging the plasma concentration of the active drug. The action of slowing down the speed of metabolism of artemisinin is an attractive feature of ketoconazole, since short half-life is one of the weaknesses of these drugs, but ketoconazole cannot be used by itself also for this very reason (Tripathi et al., 2013).

## Conclusion

**Figure 1:**
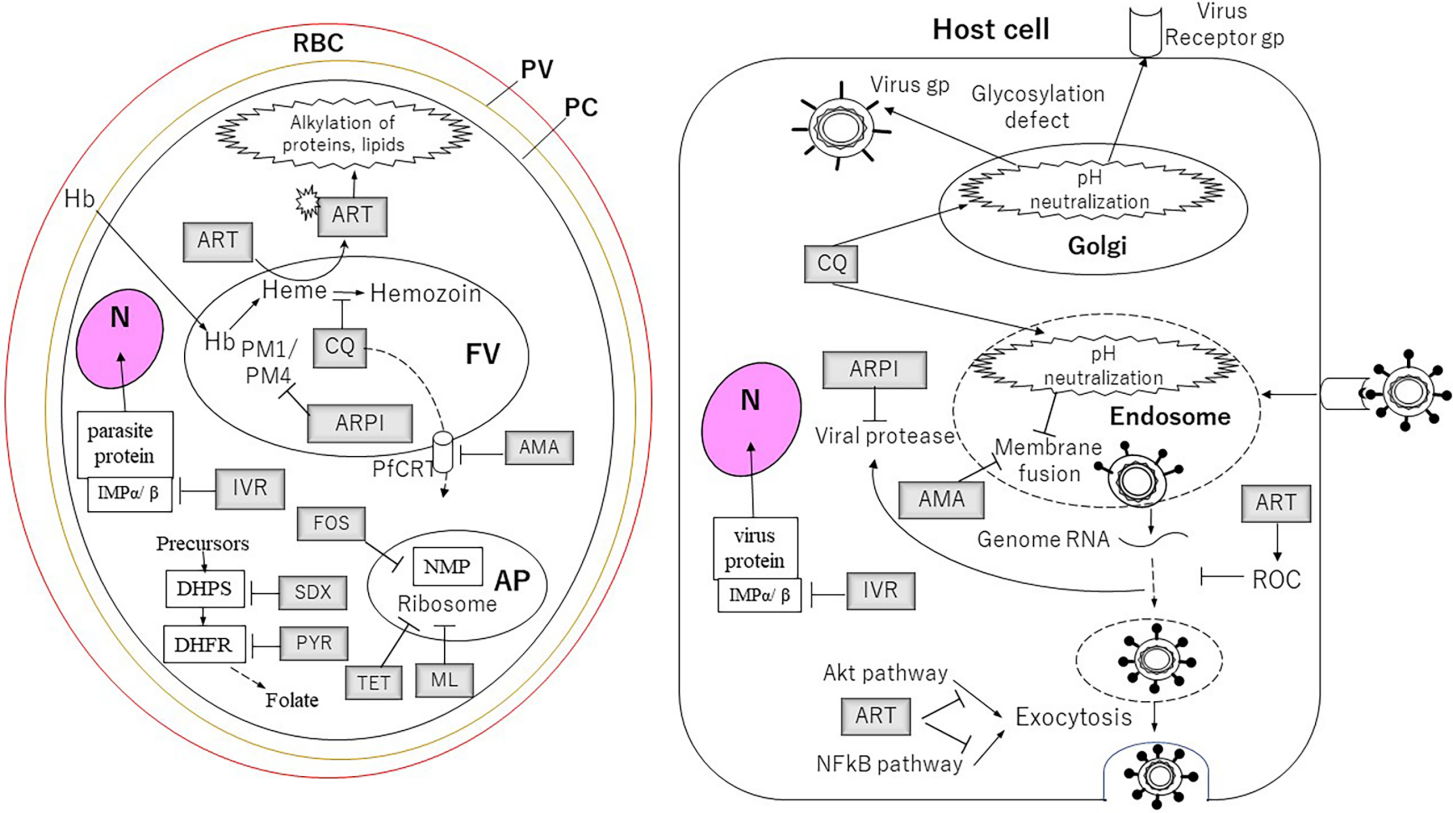
The targets of the drugs for *Plasmodium* parasites and viruses described in this article. For the cells and the organelles: RBC, red blood cell; PV, parasitophorous vacuole; PC, parasite cell; N, nucleus; FV, food vacuole; AP, apicoplast. The drugs: AMA, amantadine; ARPI, antiretroviral protease inhibitor; ART, artemisinin; CQ, chloroquine; FOS, fosmidomycin; IVR, ivermectin; ML, macrolides; PYR, pyrimethamine; SDX, sulfadoxine; TET, tetracycline. Proteins and pathway: DHFR, dihydrofolate reductase; DHPS, dihydropteroate synthase; Hb, hemoglobin; IMP, importin; NMP, non-mevalonate pathway; PM1, plasmepsin 1; PM4, plasmepsin 4.

Due to the impact of COVID-19, significant effort has been taken for the discovery of antiviral drugs since the need for serious countermeasures was recognized when the virus was spreading throughout the world. At the same time, antibacterial, antifungal, or antiparasitic drugs with promising antiviral activities were sought more than ever. The drugs described in this article are summarized in **Table 1** and **Figure 1**. The use of some major antimalarial and antiparasitic drugs such as dihydrochloroquine, artemisinin, and ivermectin has been considered for COVID-19 treatment, although none of these were proven for their effectiveness or officially approved for clinical use against the disease. On the other hand, antimalarial activities of some known antimicrobials were examined, although none of them were considered for clinical use except some antibiotics, such as sulfadoxine–pyrimethamine prophylactic treatment for pregnant women and infants. Although further studies are necessary for the drugs described in this article to find the precise potentials of these drugs, a substantial number of drugs have demonstrated significantly different mechanisms of action from the ones that existed before. Studies of repurposing drugs for different pathogens are worth investing in this sense, in addition to the financial benefit for drug-developing companies.

**Table 1:** Summary of multipurpose drugs described in this article.

Drug	Original Target	Proposed Additional Target	Proposed Mechanism of Action
Chloroquine and analogs	*Plasmodium* spp.	HIV	Inhibition of hemoglobin digestion (*Plasmodium* spp.)
		Dengue virus	Neutralization of the acidic organelles of the host cells (viruses)
		Zika virus	Alteration of glycosylation of host receptors (viruses)
		Chikungunya virus	
		Hepatitis B and C viruses	
		Ebola virus	
		SARS-CoV	
		MERS-CoV	
		SARS-CoV-2	
Artemisin and analogs	*Plasmodium* spp.	HIV	Alkylation of the cellular proteins (*Plasmodium* spp.)
		Human cytomegalovirus	Induction of reactive oxygen species (hepatitis C virus)
		Hepatitis B virus	Inhibition of NF-kB pathway (human cytomegalovirus)
		Hepatitis C virus	Inhibition of Akt pathway (human cytomegalovirus, SARS-CoV-2)
		SARS-CoV-2	
Ivermectin	Nematodes	*Plasmodium* spp.	Inhibition of importin α/β1-mediated nuclear import (*Plasmodium* spp. and viruses)
		HIV	
		Flaviviruses	
		Venezuelan equine encephalitis virus	
		Influenza A virus	
		SARS-CoV-2	
Antiretroviral protease inhibitors	HIV	*Plasmodium* spp.	Inhibition of viral aspartic protease (HIV)
			Inhibition of plasmepsins and homologs (*Plasmodium* spp.)
Amantadine	Influenza A virus	*Plasmodium falciparum*	Inhibition of M2 proton channel (influenza A)
			Unknown target in the parasite cytoplasm (*P. falciparum*)
Folate synthesis pathway inhibitors	Bacteria	*Plasmodium* spp. (trimethoprim-sulfamethoxazole)	Inhibition of folate synthesis (bacteria, *Plasmodium* spp.)
	*Plasmodium* spp. (sulfadoxine-pyrimethamine; SP)		
Tetracyclines	Bacteria	*Plasmodium* spp. (newer generations of tetracyclines)	30S ribosomal proteins and 16S rRNA (bacteria)
	*Plasmodium* spp.		ribosomal components in apicoplast (*Plasmodium* spp.)
Fosmidomycin	Bacteria	*Plasmodium* spp.	Non-mevalonate pathway of isoprenoid synthesis (bacteria, *Plasmodium* spp.)
Macrolides	Bacteria	*Plasmodium* spp.	50s ribosomal subunit (bacteria)
			Ribosomal components in apicoplast (*Plasmodium* spp.)
Manzamines	Antitumor	*Plasmodium* spp.	Glycogen synthase kinase-3 (*Plasmodium* spp.)
Marine isonitriles	*Plasmodium* spp.		Heme detoxication
Clotrimazole	Fungi	*Plasmodium* spp.	Ergosterol synthesis (fungi)
			Inhibition of hemoperoxidase (*Plasmodium* spp.)
Griseofulvin	Dermatophyte fungi	*Plasmodium falciparum*	Inhibition of mitosis by binding to microtubules (dermatophyte fungi)
			Inhibition of ferrochelatase and subsequent heme synthesis (*Plasmodium falciparum*)
Ketoconazole	Fungi	*Plasmodium* spp. (in combination with α/β arteether)	Ergosterol synthesis (fungi)
			Slowing down the drug metabolism by cytochrome P450 3A4 inhibition

## Author Contributions

TF: manuscript writing, editing, and figure and table production. TE, HT, and IS: manuscript writing. All authors contributed to the article and approved the submitted version.

## Funding

This study was supported by Japan Society for the Promotion of Science, Grant-in Aid for Scientific Program 20K06389.

## Conflict of Interest

The authors declare that the research was conducted in the absence of any commercial or financial relationships that could be construed as a potential conflict of interest.

## Publisher’s Note

All claims expressed in this article are solely those of the authors and do not necessarily represent those of their affiliated organizations, or those of the publisher, the editors and the reviewers. Any product that may be evaluated in this article, or claim that may be made by its manufacturer, is not guaranteed or endorsed by the publisher.
